# Efficacy of Azithromycin in Treatment of Acne Vulgaris: A Mini Review

**DOI:** 10.29252/wjps.8.2.127

**Published:** 2019-05

**Authors:** Sina Kardeh, Nasrin Saki, Farideh Jowkar, Bahareh Kardeh, Seyed Arman Moein, Mohammad Hossein Khorraminejad-Shirazi

**Affiliations:** 1 Cellular and Molecular Medicine Student Research Group, School of Medicine, Shiraz University of Medical Science, Shiraz, Iran;; 2 Molecular Dermatology Research Center, Department of Dermatology, Shiraz University of Medical Sciences, Shiraz, Iran;; 3 Student Research Committee, Shiraz University of Medical Sciences, Shiraz, Iran;; 4 Burn and Wound Healing Research Center, Division of Plastic and Reconstructive Surgery, Department of Surgery, Shiraz University of Medical Sciences, Shiraz, Iran;; 5 Bone and Joint Diseases Research Center, Clinical Neurology Research Center, Shiraz University of Medical Sciences, Shiraz, Iran

**Keywords:** Azithromycin, Tetracycline, Doxycycline, Acne vulgaris, Treatment

## Abstract

**BACKGROUND:**

Antibiotics are commonly used in the treatment of acne vulgaris. Considering the rise of antibiotic resistance, alternative medications may be used in the main anti-acne armamentarium. The aim of this study was to investigate the efficacy of oral azithromycin in the treatment of acne vulgaris.

**METHODS:**

Database searches were performed in PubMed and Scopus using the keywords “azithromycin” and “acne”.

**RESULTS:**

Azithromycin 500 mg once daily for 3 days per week or in cycles of 10 days for 12 weeks are the most commonly used regimens.

**CONCLUSION:**

Available experimental data suggest that oral azithromycin is an effective and well-tolerated option for treatment of acne vulgaris.

## INTRODUCTION

As a chronic inflammatory disorder of the skin, acne vulgaris affects almost 90% of adolescents, while an increasing number of adults are suffering from this disease.^1^^,^^2^ Furthermore, with an accelerated onset of puberty, the prevalence is also showing a rising trend among children.^1^ Besides the widespread use of conservative management protocols such as controlling dietary factors^3^ and face-washing,^4^ oral antibiotic therapy remains the first line of treatment for acne patients who are afflicted with physical and psychosocial side effects of moderate to severe forms of this skin condition.^5^

Tetracyclines, including tetracycline, doxycycline, minocycline and lymecycline, as well as drugs like erythromycin, clindamycin, co-trimoxazole and trimethoprim have been shown to be effective oral agents.^6^^,^^7^ Macrolide antibiotics have a substantial cumulative effect in many tissues especially epithelial lining fluid and host defense cells, such as macrophages and polymorphonuclear leukocytes.^8^ Macrolides share mild to moderate side effects such as nausea, vomiting, diarrhea, and abdominal pain, which are usually observed in erythromycin administration.^9^ Azalides like azithromycin, as a class of macrolides, possess advantageous pharmacokinetic and pharmacodynamic properties compared to other macrolides.^9^

Delivery to the infection site by phagocytes and fibroblasts results in higher concentration levels of the drug in tissues compared to serum level.^10^^,^^11^ This improves the safety and efficacy of azithromycin, which in turn not only reduces the dosage, but also decreases the frequency of drug use.^10^^,^^11^ Another pharmacological advantage of azithromycin is metabolization via hepatic pathways other than cytochrome P450, which lowers the risk of drug interactions.^10^ A bioavailability of 37% after a single 500 mg oral dose and a half-life of 2.3 to 3.2 days depending on the tissues have been reported for azithromycin.^11^

Due to the remarkable pharmacokinetics and efficacy, azithromycin is well established as a potent treatment for skin infections in adult and pediatric patients.^12^ In dermatology, clinical uses of azithromycin are not solely limited to infectious diseases. In addition to the antibacterial effects, due to the immunomodulatory and anti-inflammatory potentials of this agent, it appears that azithromycin can be administered to patients with dermatological disorders including intractable rosacea,^13^^-^^15^ psoriasis^16^^,^^17^ and synovitis, acne, pustulosis, hyperostosis, osteitis (SAPHO) syndrome.^18^^-^^20^ In this article, we reviewed the current clinical studies of azithromycin in the treatment of acne and discuss the tolerability and efficacy of this drug compared to other antibiotic options.

## MATERIALS AND METHODS

The main keywords were “azithromycin” and “acne”. English literature was searched for clinical trials using scientific search engines including Scopus and PubMed. No time limits were specified up to the date of the search (July 2018). A total of 22 articles, dating from 1994 to 2014 were found and full text of each paper was reviewed. These studies had predominantly focused on the dosage regimens, duration, and efficacy of oral azithromycin in the treatment of acne vulgaris. 

## RESULTS

Articles were summarized in Table 1 for easier comparison. The studies were conducted in various countries and differred in methodology. Study design, grouping, treatment dosage, duration and main results were described for each article.

**Table 1:** Comparison of different studies conducted in various countries regarding study design, grouping, treatment dosage, duration and main results

**Author (Year)**	Study Design*	**Grouping and Dosage**	**Main results**
Ullah *et al.*, 2014 (21)	Randomized, 386 patients, 12 weeks	Group 1: Azithromycin 500 mg/day, 4 consecutive days monthly; Group 2: Doxycycline 100 mg/day	25.9% response in azithromycin group, 66.8% response in doxycycline group, Doxycycline was a better option for acne treatment with a significant difference.
Rassai *et al.*, 2013 (22)	Investigator-blind, randomized, 148 patients, 8 weeks	Group 1: Azithromycin 500 mg /day, 3 days a week plus oral Levamisole 150 mg /day, 2 days a week; Group 2: Azithromycin 500 mg/day, 3 days a week	Azithromycin plus levamisole was significantly more effective than azithromycin alone in reducing inflammatory acne lesions.
Hasibur *et al.*, 2013 (23)	Open-label, non- comparative, 82 patients, 24 weeks	Pulsed oral Azithromycin: 500 mg/day on 3 consecutive days in each week for 1 month with low-dose Isotretinoin: 0.3 mg/kg/day for 6 month	Complete cure in 80 (97.56%) patients, Low-dose isotretinoin plus oral azithromycin pulse can be effective in moderate to severe acne.
Moravvej *et al.*, 2012 (24)	Investigator-blind, randomized, 60 patients, 12 weeks	Group 1: Azithromycin 500 mg/day three times a week; Group 2: Doxycycline 100 mg/day. All patients used topical tretinoin cream every other night.	Both groups showed significant and similar improvements in inflammatory lesion count with mild and transient side effects.
Kayhan *et al.*, 2012 (25)	Randomized, 60 patients, 12 weeks	Group 1: oral Azithromycin 500 mg/day on 3 consecutive days followed by 7 days rest (a 10-day cycle); Group 2: Doxycycline 100 mg/day. Topical adapalene gel was added to the systemic treatment in both groups.	Both treatments were safe and effective with significant and similar improvement in the quality of life scale scores and minimal side effects.
Babaeinejad *et al.*, 2011 (26)	Double-blind, randomized, 100 patients, 12 weeks	Group 1: Azithromycin: 500 mg/day, on 4 consecutive days per month; Group 2: Doxycycline: 100 mg/day	Both antibiotics were effective with minor complications the in treatment of moderate acne. Doxycycline was significantly more effective in patients above 18 years.
De *et al.*, 2011 (27)	Open-label, non- comparative, 66 patients, 16 weeks	Combination of low-dose Isotretinoin 0.3 mg/kg/day and pulsed oral azithromycin 500 mg/day on 3 consecutive days every 2 weeks	93.9% complete clearance and 11.3% disease relapse, The combination of low-dose isotretinoin and oral azithromycin was an effective treatment for severe acne with acceptable adverse-effects.
Maleszka *et al.*, 2011 (28)	Double-blind, randomized, 240 patients, 12 weeks	Group 1: Azithromycin 500 mg/day for 3 days in the first week, followed by 500 mg tablets weekly to complete 10 weeks of treatment; Group 2: 2 Doxycycline 100 mg capsules on the first day, then once a day during 12 weeks of treatment	Similar reduction in number of lesions with both azithromycin and doxycycline, No difference was observed in the incidence of side effects between the two treatment groups.
Antonio *et al.*, 2008 (29)	Open-label, non- comparative, randomized, 57 patients, 12 weeks	Azithromycin: 500 mg on 3 consecutive days with intervals of 7 days without medication	Azithromycin was well tolerated with a significant reduction in the number of lesions. The majority of adverse effects were related to the gastrointestinal and central nervous systems.
Innocenzi *et al.*, 2008 (30)	Open-label, non- comparative, 46 patients, 12 weeks	Azithromycin: 500 mg thrice weekly for 12 weeks. Plus: 0.1% topical adapalene (gel or cream) once daily in the evening, and benzoyl peroxide (gel) once daily in the morning.	Significant improvement and reduction in lesions, Safe and effective treatment regimen for moderate acne with excellent patient compliance. Reported side effects were diarrhea and abdominal pain.
Wahab *et al.*, 2008 (31)	Randomized, 60 patients,12 and 20 weeks	Group 1: Isotretinoin: 0.5-1 mg/kg for 5 months; Group 2: Azithromycin: 500 mg 3 days a week for 3 months. Topical adjuvant therapy e.g. erythromycin lotion initially and then adapalene was given in both the groups.	Both treatments were useful for moderate and severe acne. Isotretinoin appeared to be superior to weekly pulse dose of azithromycin. Mild nausea and abdominal discomfort were reported in Azithromycin group.
Bardazzi *et al.*, 2007 (32)	Open-label, non-comparative, 52 patients, 8 weeks	Azithromycin: 500 mg thrice weekly	Remarkable improvement in 90.4% of patients, Safe and effective treatment regimen for acne in adolescents, with excellent patient compliance, Gastrointestinal intolerance was reported by three patients (5.8%).
Basta-Juzbašić *et al.*, 2007 (33)	Open-label, randomized, 93 patients, 24 weeks	3 dosage regimens of azithromycin: Group 1: 4.5 g total dose in 7 weeks; Group 2: 6.0 g total dose in 10 weeks; Group 3: 7.5 g total dose in 13 weeks, A 3-day course of 500 mg/day followed by 500 mg/week for another 6 weeks in group 1, 9 weeks in group 2, and 12 weeks in group 3. Subjects were allowed to apply a keratolytic lotion topically twice a day.	Cure rate: 36.11% in group 1, 58.82% in group 2 and 55.88% in group 3. Azithromycin in a total dose of 6.0 g in 10 weeks was beneficial in the treatment of papulopustular acne with few side effects and good patient compliance.
Ghoshal *et al.*, 2007 (34)	Randomized, 61 patients, 12 weeks	Group 1: topical adapalene (0.1%) gel once daily at bedtime and 1 FTU for the entire face; Group 2: 500 mg oral azithromycin for 3 consecutive days in a week; Group 3: combination of the two therapies. Patients washed their face with soap for three to four times a day.	Although combination therapy showed highest reduction in the number of inflammatory lesions, there was no significant difference in the efficacy of the three treatment groups.
Naieni *et al.*, 2006 (35)	Investigator-blind, randomized, 58 patients, 12 weeks	Three different Azithromycin regimens: Group 1: 5 consecutive days, 500 mg on the first day and 250 mg/day for another 4 days per month; Group 2: 500 mg/day for 4 consecutive days per month; Group 3: 250 mg/day thrice weekly.	Low dose azithromycin was as effective as a high dose with more compliance and fewer side-effects. Diarrhea was the only complication in three patients of group 3.
Rafiei *et al.*, 2006 (36)	Investigator-blind, randomized, 290 patients, 12 weeks	Group 1: Azithromycin 500 mg for 3 consecutive days a week for 1 month, then 250 mg every other day for the following 2 months; Group 2: Tetracycline 1 g with similar protocol	Azithromycin response (84.7%) was slightly higher in reducing inflammatory lesions compared with tetracycline (79.7%). Similar rate of GI side effects (11%) were reported in both groups.
Kus *et al.*, 2005 (37)	Investigator-blind, randomized, 45 patients, 20 weeks	Group 1: Azithromycin 500 mg/day on 3 consecutive days per week in the first, on 2 consecutive days per week in the second, and on 1 day per week in the third month; Group 2: Doxycycline 100 mg twice a day for the first month and once a day for the second and third months	Significant and similar improvement of acne lesions in both drugs,
Kapadia *et al.*, 2004 (38)	Open-label, non- comparative, 35 patients, 12 weeks	Azithromycin 500 mg orally thrice weekly for 12 weeks, 0.05% tretinoin cream was applied only to the face	Remarkable improvement in 82.9% of patients in the first 4 weeks, Adverse events were reported by 11.4% of patients. Azithromycin was a safe and effective treatment for acne vulgaris with excellent patient compliance.
Singhi *et al.*, 2003 (39)	Non-randomized, 62 patients, 12 weeks	Group 1: azithromycin was administered 500 mg daily for 3 consecutive days in a 10-day cycle, with seven drug-free days in each cycle; Group 2: doxycycline 100 mg daily. Topical erythromycin was prescribed to all patients	77.26% improvement in azithromycin group and 63.74% in the doxycycline group, The combination of azithromycin with topical erythromycin was significantly better with lower side effects compared to doxycycline with topical erythromycin.
Parsad *D et al.*, 2001 (40)	Randomized, 50 patients, 12 weeks	Group 1: Doxycycline 100 mg/day; Group 2: Azithromycin 500 mg/day for 4 days per month. Topical 0.05% Tretinoin cream was prescribed to all patients.	A monthly dose of azithromycin was as effective as daily doxycycline.
Fernandez-Obregon, 2000 (41)	Retrospective, 79 patients, 10 weeks	Individuals that were unable to tolerate tetracycline, erythromycin, minocycline, and doxycycline, were treated with azithromycin 250 mg three times a week. Most patients also used topical care.	Significant improvement was noted in 4 weeks in all agents, while azithromycin was significantly better with a greater than 80% reduction in inflammatory acne lesions (85.7%) vs. an average of 77.1% for all other agents.
Gruber *et al.*, 1998 (42)	Open-label, 72 patients, 6 weeks	Group 1: oral azithromycin 500 mg for 4 days every 10 days, for a total of four cycles; Group 2: minocycline 100 mg/day for 6 weeks	75.8% treatmentresponse with azithromycin and 70.5% with minocycline; Azithromycin was at least as effective as minocycline in the treatment of comedonic and papulopustular acne with well and similar tolerance.

## DISCUSSION

For a long time, it has been believed that administration of macrolides such as azithromycin has anti-inflammatory effects.^43^ In 2000, Ianaro *et al.* demonstrated that macrolides can suppress inflammation by inhibiting production of proinflammatory molecules such as PGE_2_, TNF-α, and NO_x._^44^ In addition, it has been observed that macrolides down-regulate neutrophil migration, ROS production and apoptosis. There is also growing evidence that macrolides, particularly azithromycin, exert immunomodulatory effects by diminishing production of IL-1α and IL-8 cytokines.^9^^,^^45^

Moreover, upregulated expression of proinflammatory factors such as IL-1α, TNF-α, PGE_2_, and IL-8 has been observed in acne patients.^46^^-^^49^ Thus, the therapeutic effects of azithromycin in acne vulgaris patients might be mediated by antimicrobial aspects of this agent as well as anti-inflammatory and immunomodulatory potentials.^50^ Many studies focused on the comparison of azithromycin and doxycycline, suggesting significant improvement by using each of these medications with no remarkable differences in treatment results.^24^^-^^26^^,^^28^^,^^37^^,^^40^ However, the study by Ullah *et al.* on 386 patients showed that doxycycline was more effective.^21^

In addition, Babaeinejad *et al.* concluded that doxycycline is more effective in patients above 18 years old.^26^ It should also be noted that in both these studies, patients were treated with 4 consecutive days of 500 mg azithromycin per month; while it seems that intermittent higher doses,^24^^,^^25^^,^^39^ mainly three times in 10-days or thrice-weekly, may be more advantageous. Further, administration of azithromycin in combination with topical erythromycin results in significantly better improvement than doxycycline combined with topical erythromycin.^39^ Also, no beneficial effects of minocycline were observed when compared to azithromycin.^42^

A study by Rafiei *et al.* found azithromycin to have a slightly higher efficacy in the treatment of inflammatory acne lesions in comparison to tetracycline.^36^ On the other hand, retrospective study of patients who could not tolerate tetracycline, erythromycin, minocycline, and doxycycline proved that azithromycin is a significantly better antibiotic regimen for acne.^41^ Despite the usefulness of both isotretinoin and azithromycin in the treatment of moderate to severe acne, superior efficacy of isotretinoin is evident.^31^

To enhance treatment outcome, various studies have utilized adjuvant drugs in combination therapies including topical tretinoin, adapalene, benzoyl peroxide and erythromycin. The combination of oral azithromycin with either topical adapalene^34^ or oral levamisole^22^ provided more efficacious treatment than azithromycin alone. In combination with isotretinoin, two studies have reported cure rates above 90%.^23^^,^^27^ Also, combined with adapalene plus benzoyl peroxidase, azithromycin was indicated as a safe option.^30^ Considering azithromycin dosing for acne treatment, the most commonly used strategies are 3 consecutive days of 500 mg azithromycin in 10 days for 12 weeks,^25^^,^^29^^,^^39^ azithromycin 500 mg three times per week for 8^22^^,^^32^ or 12 weeks^24^^,^^30^^,^^31^^,^^34^^,^^38^ and also 4 consecutive days per month continued for 12 weeks.^21^^,^^26^^,^^35^

In general, azithromycin was considered to be tolerable with low adverse effects including GI (namely nausea, diarrhea and abdominal pain)^29^^-^^32^^,^^35^^,^^36^ and CNS symptoms.^29^ With regard to tetracycline induced photosensitivity, use of azithromycin may also be beneficial in summer months.^37^ Low side effects and clinical tolerance along with convenient consumption have resulted in good patient compliance with azithromycin.^25^^,^^28^^,^^32^^,^^35^^,^^37^^-^^39^^,^^41^^,^^42^ Various advantages have been suggested for azithromycin compared with other macrolides. There has always been a debate regarding the administration of antibiotics to pregnant and lactating patients due to possible side effects on fetus and infant.^51^

In spite of the potential risks of azithromycin crossing placental barrier, no adverse effects have been observed in fetal development in animal models. Furthermore, FDA has classified azithromycin as a B category drug for treatment of acne vulgaris, confirming the compatibility of this drug with pregnancy.^51^^,^^52^ Although azithromycin has the longest half-life among macrolides and can be transferred to breast milk, it has not shown toxicity to fetus or infant.^53^ Moreover, studies have indicated that administration of azithromycin to pregnant patients is as efficient as doxycycline in non-pregnant acne vulgaris patients.^37^ Overall, these studies approved the use of azithromycin for the treatment of lactating or pregnant acne vulgaris patients.

Given the differences in azithromycin dosing and timing of administration, the conclusion on a specific effective therapeutic regimen for acne vulgaris remains indefinite. Nonetheless, most studies applied azithromycin thrice weekly or in 10 days with successful results. With regards to low incidence and mild side effects and also potential anti-inflammatory and immunomodulatory effects of azithromycin, this agent is a good choice for those who cannot tolerate other commonly used oral antibiotics. It is also important to consider that azithromycin is a safe drug for lactating and pregnant women suffering from acne vulgaris, making this drug a promising treatment. Furthermore, no resistance has been yet reported regarding treatment of acne vulgaris with azithromycin. 

## CONFLICT OF INTEREST

The authors declare no conflict of interest.
